# Novel Small Molecule Inhibitors of Protein Kinase D Suppress NF-kappaB Activation and Attenuate the Severity of Rat Cerulein Pancreatitis

**DOI:** 10.3389/fphys.2017.01014

**Published:** 2017-12-07

**Authors:** Jingzhen Yuan, Tanya Tan, Meng Geng, Grace Tan, Chintan Chheda, Stephen J. Pandol

**Affiliations:** ^1^Cedars-Sinai Medical Center, Los Angeles, CA, United States; ^2^Veterans Affairs Greater Los Angeles Healthcare System, Department of Medicine, University of California, Los Angeles, Los Angeles, CA, United States; ^3^Georgetown University Medical Center, Washington, DC, United States; ^4^Frank Netter H. School of Medicine at Quinnipiac University, Hamden, CT, United States; ^5^Vanderbilt University, Nashville, TN, United States

**Keywords:** NF-κB, protein kinase D, PKD inhibitors, pancreatitis

## Abstract

Nuclear factor-kappa B (NF-κB) activation is a key early signal regulating inflammatory and cell death responses in acute pancreatitis. Our previous *in vitro* studies with molecular approaches on AR42J cell showed that protein kinase D (PKD/PKD1) activation was required in NF-κB activation induced by cholecystokinin 8 (CCK) or carbachol (CCh) in pancreatic acinar cells. Recently developed small molecule PKD inhibitors, CID755673 and CRT0066101, provide potentially important pharmacological approaches to further investigate the effect of PKD in pancreatitis therapy. The aim of this study was to explore whether CID755673 and CRT0066101 block NF-κB activation with *in vitro* and *in vivo* models of experimental pancreatitis and whether the small molecule PKD inhibitors have therapeutic effects when given before or after the initiation of experimental pancreatitis. Freshly prepared pancreatic acini were incubated with CID755673 or CRT006101, followed by hyperstimulation with CCK or CCh. For *in vivo* experimental pancreatitis, rats were treated with intraperitoneal injection of CID755673 or CRT0066101 prior to or after administering cerulein or saline. PKD activation and NF-κB-DNA binding activity in nuclear extracts from pancreatic acini and tissue were measured. The effects of PKD inhibitors on pancreatitis responses were evaluated. Our results showed that both CID755673 or CRT0066101 selectively and specifically inhibited PKD without effects on related protein kinase Cs. Inhibition of PKD resulted in significantly attenuation of NF-κB activation in both *in vitro* and *in vivo* models of experimental pancreatitis. NF-κB inhibition by CID755673 was associated with decreased inflammatory responses and attenuated severity of the disease, which were indicated by less inflammatory cell infiltration, reduced pancreatic interleukin-6 (IL-6) and monocyte chemoattractant protein-1 (MCP-1), decreased intrapancreatic trypsin activation, and alleviation in pancreatic necrosis, edema and vacuolization. Furthermore, PKD inhibitor CID755673, given after the initiation of pancreatitis in experimental rat model, significantly attenuated the severity of acute pancreatitis. Therapies for acute pancreatitis are limited. Our results indicate that small chemical PKD inhibitors have significant potential as therapeutic interventions by suppressing NF-κB activation.

## Introduction

Acute pancreatitis is a disorder with pathologic features of inflammation and necrosis in pancreatic tissue. Increasing evidence has demonstrated that inflammatory response plays a pivotal role in the development of the disease (Grady et al., 1997; Frossard et al., 1999; Bhatia et al., 2000; Gukovskaya et al., 2002; Pandol, 2006; Pandol and Raraty, 2007). The nuclear transcription factor NF-κB, a key signal triggered during pancreatitis, regulates the expression of pro-inflammatory cytokines, chemokines, immune receptors, and other inflammatory molecules (Gukovsky et al., 1998; Frossard et al., 1999; Tak and Firestein, 2001; Vaquero et al., 2001), which are responsible for the severe systemic inflammatory complications of the disease (Grady et al., 1997; Frossard et al., 1999; Satoh et al., 1999; Bhatia et al., 2000; Vaquero et al., 2001; Chen et al., 2002). Multiple experimental models of pancreatitis have shown that NF-κB activation in acinar cells is one of earliest events in acute pancreatitis and the inhibition of NF-κB activation alleviates the severity of the disease (Gukovsky et al., 1998, 2003; Satoh et al., 1999; Vaquero et al., 2001). Further, NF-κB activation within pancreas directly triggered by adenovirus-mediated gene transfer can initialize pancreatic and systemic inflammatory responses (Chen et al., 2002). Therefore, development of pharmacological interventions to suppress NF-κB activation is necessary to prevent severe pancreatitis in early stage of the disease.

Serine/threonine protein kinase D (PKD) family, composed of PKD/PKD1, PKD2, and PKD3, has recently characterized as an important target in the signaling cascades initiated and transduced through G protein coupled receptors, phospholipase C, second messengers, and PKC-dependent or -independent mechanisms in various cell types including pancreatic acinar cells (Rozengurt et al., 2005; Berna et al., 2007; Yuan et al., 2008; Chen et al., 2009; Rozengurt, 2011). PKD family members have been implicated in the regulation of numerous cell biological functions, such as Golgi complex integrity and protein secretion (Rozengurt et al., 2005; Fugmann et al., 2007; Li et al., 2008; Rozengurt, 2011), phosphorylation of heat shock protein (Yuan and Rozengurt, 2008) and histone deacetylase (Matthews et al., 2006; Sinnett-Smith et al., 2014), cell apoptosis (Trauzold et al., 2003), and proliferation (Rozengurt et al., 2005), oxidative stress and inflammation, heart diseases and malignant tumors (Storz et al., 2004a; Chiu et al., 2007; Fielitz et al., 2008; Sharlow et al., 2008; Harikumar et al., 2010). Recent studies has revealed that PKDs are key transcriptional regulators through induction of nuclear transcription factors c-jun (Hurd et al., 2002), the cAMP-response element-binding protein (Johannessen et al., 2007), and particularly, NF-κB (Storz et al., 2004a,b).

PKDs can be activated by a numbers of gastrointestinal secretagogues in pancreatic acini (Berna et al., 2007; Yuan et al., 2008, 2012; Chen et al., 2009; Thrower et al., 2011; Yuan and Pandol, 2016). Our studies showed that the peptide hormone cholecystokinin 8 (CCK) and the cholinergic agonist carbachol (CCh) induced a dose-dependent rapid activation of PKD in pancreatic acini, which was closely correlated with increased NF-κB activity (Yuan et al., 2008). With a molecular approach to up- or down-regulate PKD expression in AR42J acinar cell line, we showed that PKD mediated NF-κB activation caused by CCK or CCh in pancreatic acinar cells (Yuan et al., 2008).

CRT0066101 and CID755673, two small molecule chemicals, have been newly identified as PKD specific inhibitors. Two research groups have reported the *in vitro* and *in vivo* anti-tumor growth effect of the inhibitors in pancreatic ductal adenocarcinoma and prostate cancer respectively (Sharlow et al., 2008; Harikumar et al., 2010). Of significant importance for pancreatitis, we have reported that CRT0066101 reduces secretagogues-induced zymogen premature activation in primary pancreatic acini (Thrower et al., 2011) and that CID755673 treatment attenuates pancreatic necrotic death in cerulein-induced experimental pancreatitis models (Yuan et al., 2012; Yuan and Pandol, 2016). The aims of the current study are to explore (1) whether the novel PKD inhibitors block NF-κB activation in experimental pancreatitis models, and (2) whether suppressing of NF-κB activation by the PKD inhibitors is associated with attenuation of inflammatory response and severity of pancreatitis, as well as (3) the therapeutic benefit of the PKD inhibitors administered after induction of the pancreatitis.

Our results identified PKD as a novel early signaling triggered through CCK or cholinergic receptor to mediate NF-κB activation in acute pancreatitis and demonstrated that PKD inhibitors potently blocked NF-κB activation in *in vitro* and *in vivo* experimental pancreatitis models. Importantly, NF-κB inhibition by the PKD inhibitor CID755673 was associated with significantly decreased inflammatory responses and alleviated pancreatic histopathologic changes in pancreatitis. The beneficial effects in pancreatitis were present both when the PKD inhibitor was given before initiation of pancreatitis and during pancreatitis.

Our studies indicate that the small chemical PKD suppressors possess significant potential as therapeutic intervention to alleviate/prevent serious pancreatitis at early stage of the disease or to prevent recurrent pancreatitis through suppressing NF-κB activation.

## Materials and methods

### Reagents

CCK was from American Peptide (Sunnyvale, CA); Medium 199 was from GIBCO (Grand Island, NY). ATP and [γ-32P] ATP were from Perkin Elmer (Torrance, CA). CRT0066101 and CID755673 were obtained from TOCRIS (Mo, USA). Nitrocellulose membranes were from Schleicher and Schuell BioSience. Carbachol and GF1 (also known as GF109203X or bisindolylmaleimide I) were from Calbiochem (La Jolla, CA). Antibodies against PKD C-20, IκB-α, or LDH were from Santa Cruz Biotechnology (Santa Cruz, CA). Phosphoserine 744/748 PKD antibody that detects primarily the phosphorylated state of Ser 744 (Jacamo et al., 2008), phosphoserine 916 PKD antibody, antibodies for NF-κB P65, phosphoserine 32/36 IκB-α, GAPDH, ERK1/2 were obtained from Cell Signaling Technology (Beverly, MA). IL-6 antibody was from PeproTech (Rocky Hill, NJ) and MCP-1 antibody was from Antibodies-Online Inc. (Secaucus, NJ). Protein-A-agarose was from Roche Applied Science (Mannheim, Germany) and PKD substrate syntide-2, was from Bachem (Chicago, IL). Other items were from standard suppliers or as indicated in text.

### Animals

Male Sprague-Dawley rats were used in all experiments. The animals were kept in a temperature-(23 ± 2°C) and humidity- (55 ± 5%) controlled room with a 12-h light/dark cycle (lights on at 07:00 a.m.). The animals were provided *ad libitum* standard rat chow and tap water.

### Animal care guidelines

Animal care and all procedures were approved by the Institutional Animal Care and Use Committees of the Veterans Affairs Greater Los Angeles Health Care System and Cedars-Sinai Medical Center, Los Angeles in accordance with the National Institutes of Health guidelines USA. Rats were kept under specific pathogen-free conditions and received humane care according to the guidelines of our institution. All experiments were performed according to the guidelines of the National Institutes of Health guidelines.

### Preparation and treatments of dispersed pancreatic acini and preparation of cell lysate

Pancreatic acinar cells were prepared from male Sprague-Dawley rats (75–100 g) using a collagenase digestion method as described previously (Gukovsky et al., 1998; Yuan et al., 2008) and then incubated in medium 199 supplemented with 0.01% trypsin inhibitor (w/v), penicillin (100 U/ml), and streptomycin (0.1 mg/ml) at 37°C in a 5% CO_2_-humidified atmosphere. For experimental purposes, the acinar cells were pre-incubated in the medium 199 for 2 h at 37°C with or without inhibitors as described previously (36) and then treated further with or without agonists. Acinar cells were collected and washed with PBS. Aliquots of the acinar cells were lysed and sonicated in lysis buffer as described previously (Satoh et al., 2004). After centrifugation at 15,000 × g for 10 min at 4°C, the protein concentration in supernatants was measured using the Bio-Rad protein assay reagent.

### Experimental pancreatitis and pancreatic tissue lysate preparation

Sprague-Dawley rats (male, 120–150 g) were pre-treated with intraperitoneal (IP) injection of PKD inhibitor CID755673 (CID, 15 mg/kg) or CRT0066101 (CRT, 10 mg/kg) 60 min prior to initiation of pancreatic stress with up to 4 hourly IP injections of cerulein (20 μg/kg), a CCK analog used for experimental pancreatitis models. Control animals received similar injections of physiologic saline. We choose the dosages of the PKD inhibitors in the animal experiments based on our previous dose response (Yuan et al., 2012). Then the animals were sacrificed in 30 min after first IP injection of cerulein or in 60 min after 4th hourly IP injection of cerulein. Blood samples and the pancreas were obtained and stored at −80°C for measurements. Portions of frozen tissue were homogenized on ice in lysis buffer as described previously (Satoh et al., 2004). After sonication the lysates were rotated for 40 min at 4°C and centrifuged at 4°C for 15 min at 16,000 g. The supernatants were collected and stored at −80°C.

To determine potential of PKD inhibitor as therapeutics after the disease has commenced, we induced rat pancreatitis by 6 hourly cerulein injections. CID755673 (15 mg/kg) or vehicle was administered by IP injection to rats at early and late stages after the initiation of pancreatitis (i.e., 30 min after 1st and 4th of IP injection of cerulein). The animals were sacrificed in 1 h after the 6 hourly cerulein injections.

### Preparation of nuclear extracts and NF-κB DNA binding activity measurement

Nuclear protein extracts were prepared using ActiveMotif Nuclear Extract Kit (Carlsbad, CA) following the manufactory instructions. NF-κB DNA binding activities were measured with electrophoretic mobility shift assay (EMSA) as described in our laboratory previously (Gukovsky et al., 1998; Yuan et al., 2008).

### Western blot analysis, PKD immunoprecipitation, and *in vitro* kinase assay

Western blot analyses were performed as described previously (Yuan et al., 2003, 2008, 2012). The membranes were blocked by 1–2 h incubation with 5% non-fat dried milk in Tris-buffered saline, pH 7.2 and probed overnight at 4°C with specific primary antibodies at a 1:500–1:1,000 dilution in the Tris-buffered saline containing 3% non-fat dried milk. Then the membranes were incubated with secondary antibodies conjugated with horseradish peroxidase at 1:5,000 dilutions for 1 h at room temperature. Blots were developed by using the enhanced chemiluminescence detection kit (Pierce). When re-probing was necessary, the membrane was stripped of bound antibody by incubating in Re-Blot Plus Mild Solution (Millipore, Temecula, CA) for 20 min.

PKD in pancreatic tissue lysates was immunoprecipitated at 4°C for 3 h with the PKD C-20 antibody (1:100) and protein-A-agarose as previously described (Yuan et al., 2002, 2003, 2008, 2012). Exogenous substrate syntide-2 phosphorylation by immunoprecipitated PKD was carried out by mixing 20 μl of the washed immunocomplexes with 10 μl of a phosphorylation mixture containing 100 μM ATP (including [γ-^32^P]ATP at 2 μCi/assay or with specific activity, 400–600 cpm/pmol) and 2.5 mg/ml syntide-2 (PLARTLSVAGLPGKK) in kinase buffer. After 10 min of incubation at 30°C, the reaction was stopped by adding 100 μl of 75 mM H_3_PO_4_, and 75 μl of the supernatant was spotted on P-81 phosphocellulose paper. Free [γ-^32^P]ATP was separated from the labeled substrate by washing the P-81 paper four times for 5 min in 75 mM H_3_PO_4_. The papers were dried, and the radioactivity incorporated into syntide-2 was determined by Cerenkov counting.

### Enzymatic assays

Animal serum amylase and lipase activities were determined by Antech Diagnostics (Irvine, CA) Custom Service. Active trypsin in pancreatic tissue was measured by using Boc-Gln-Ala-Arg-AMC as a substrate by a fluorescent assay as described previously (Gukovskaya et al., 2002).

### Quantification of necrosis

Quantification of necrosis in *in vivo* pancreatitis was performed on pancreatic tissue (collected after 4 or 6 hourly cerulein injections) sections stained with H&E as described previously (Mareninova et al., 2006; Sung et al., 2009; Yuan et al., 2012). Cells with swollen cytoplasm, loss of plasma membrane integrity, and leakage of organelles into interstitium were considered necrotic. A total of at least 2,000 acinar cells were counted on tissue sections from each animal and 3–5 animals per condition were counted.

### Histologic analysis for pancreas inflammatory cell infiltration and vacuolization and measurement of edema

Quantification of inflammatory cell infiltration and vacuolization was performed on H&E stained pancreatic tissue (4–6 hourly cerulein injections) sections from at least 4 mice per group and expressed as the number of inflammatory cells or vacuoles per 100 acinar cells. Pancreatic edema grading was made on the H&E stained tissue sections from 0 to 3 according to the Schoenberg grading system (Schoenberg et al., 1990): 0: no edema; 1: interlobular edema; 2: moderate interlobular and intra-acinar edema; and 3: severe interlobular and intra-acinar edema.

### Statistical analysis

Results were expressed as the mean ± *SD* of at least three independent experiments. The experimental data was evaluated by the analysis of variance (ANOVA) followed by Bonferroni multiple comparison *post-hoc* tests with the GraphPad Prism software (GraphPad Software Inc. La Jolla, California). *T*-tests were used to analyze differences between two groups. *P* < 0.05 was considered statistically significant.

## Results

### CID755673 and CRT0066101 inhibit both PKD activation and NF-κB activation in *in vitro* model of pancreatitis induced by a high dose of CCK or CCh stimulation

**Figure 1:**
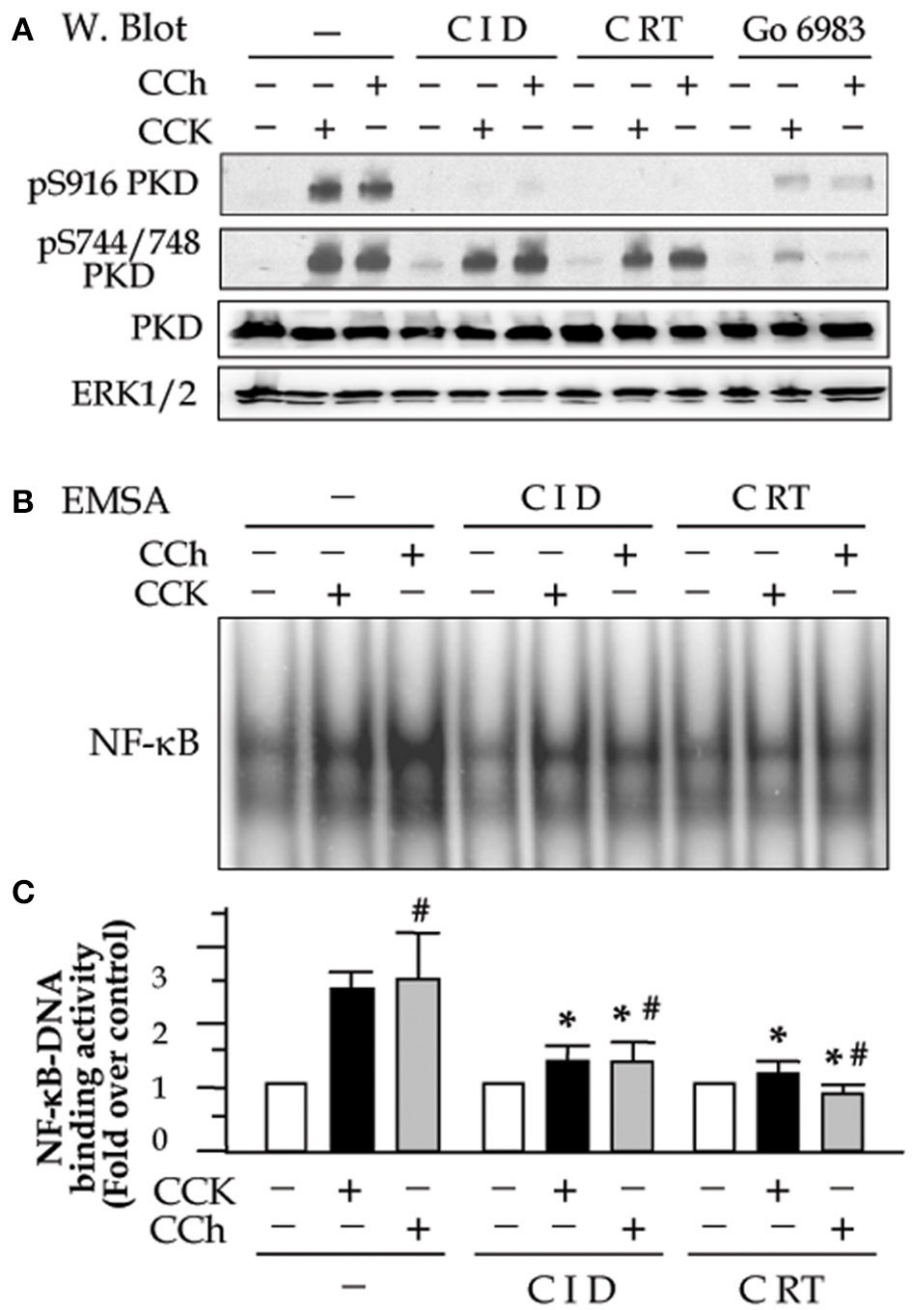
Small molecule chemicals CID755673 and CRT0066101 inhibit high dose secretagogue-induced PKD activation and NF-κB activation in pancreatic acinar cells. Rat pancreatic acinar cells were incubated with or without CID755673 (CID, 25 μM) or CRT0066101 (CRT, 10 μM) and PKC inhibitor Go6983 (10 μM) for 2 h prior to 100 nM CCK or 200 μM CCh treatment for an additional 30 min. **(A)** Western blot was performed to analyze PKD phosphorylation in cell lysate. PKD and ERK1/2: for loading controls. **(B)** NF-κB-DNA binding activity was measured in cell nuclear extracts (NE) by EMSA. **(C)** NF-κB band intensities were quantified in the PhosphorImager and normalized on the band intensity in unstimulated control acini. Values are means ± SE (*n* = 3). ^*^p < 0.05 vs. CCK or CCh (#) alone without inhibitor pre-treatments.

We previously showed that CRT0066101 reduced secretagogues-induced PKD activation (Thrower et al., 2011) and that CID755673 inhibited CCK-induced PKD activation (Yuan et al., 2012) in primary pancreatic acinar cells. Here, in a consistent experiment, we examined the effect of both inhibitors on PKD activation induced by CCK and CCh (Figure 1). Pancreatic acini freshly prepared from rat were incubated with 25 μM CID755673 or 10 μM CRT0066101 for 2 h followed by challenging with a high dose of the pancreatic secretagogues CCK (100 nM) or CCh (200 μM) which were known to induce pancreatitis pathologies in acini *in vitro* (Gukovsky et al., 2003; Yuan et al., 2008, 2012; Thrower et al., 2011). Our results showed that both CCK and CCh dramatically induced PKD activation determined by Western blot to measure autophosphorylation of PKD/PKD1 Ser916. The PKD activation was potently inhibited by pre-incubation with either CID755673 or CRT0066101. Both inhibitors did not affect PKD phosphorylation at its activation loop (Ser744/748) (Figure 1A) which was known largely via PKC-dependent pathways in pancreatic acinar cells (Berna et al., 2007; Yuan et al., 2008), but the Ser744/748 phosphorylation was almost abolished by Go6983 (10 μM), a conventional and novel PKC inhibitor (Figure 1A). The results indicate that CID755673 and CRT0066101 directly affect PKD activation independent of upstream pathways known to mediate PKD activation.

Next, to determine the effect of the PKD inhibitors on CCK- or CCh-induced NF-κB activation, we prepare nuclear extracts using the acini collected in the same experiments described above. NF-κB-DNA binding activity in the nuclear extracts was measured by EMSA. Either CID755673 or CRT0066101 significantly decreased NF-κB activation induced by CCK or CCh (Figures 1B,C). Inhibition of NF-κB was corresponding to the inhibition of PKD. These results are consistent with our previous findings achieved in AR42J cell line by using molecular approaches to upregulate or downregulate PKD expression (Yuan et al., 2008).

**Figure 2:**
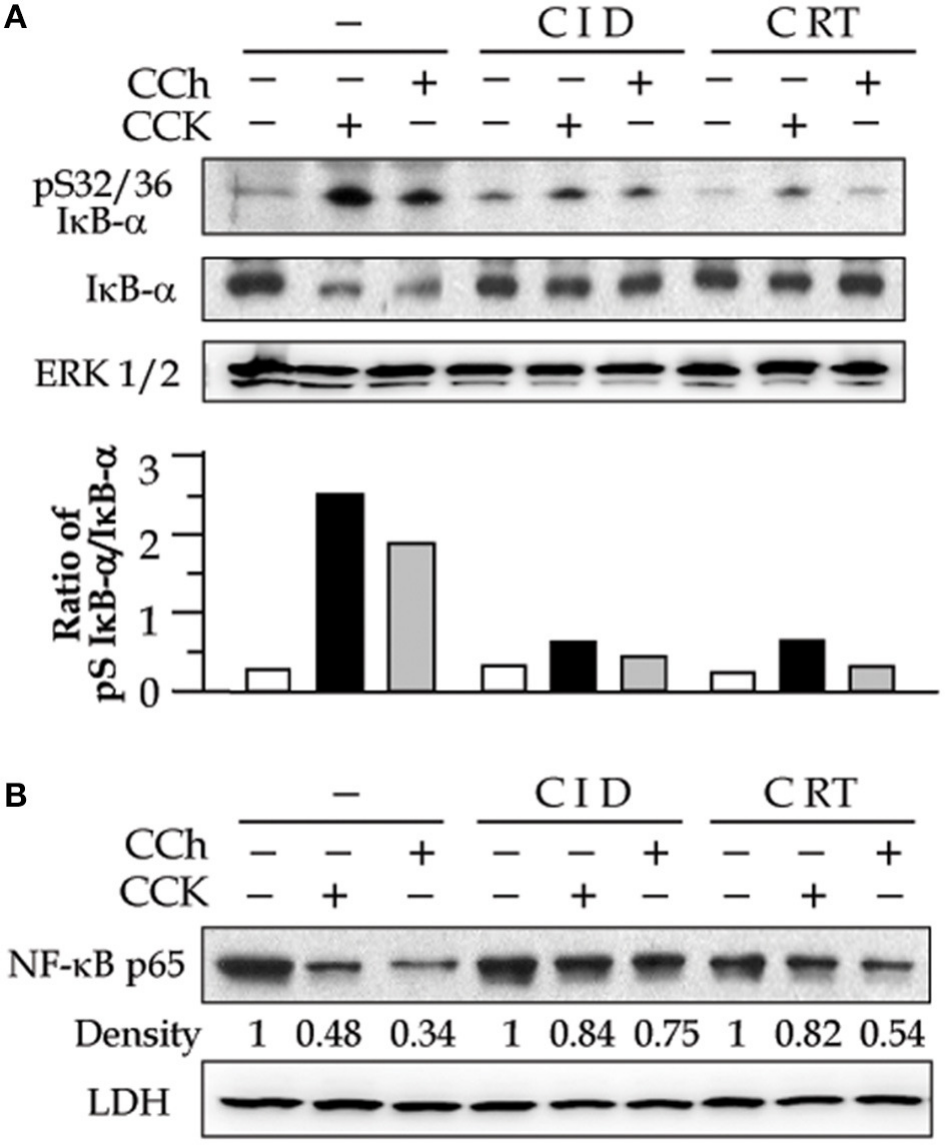
PKD inhibitors decrease secretagogue-induced IkB-α phosphorylation and degradation and nuclear translocation of NF-κB. Rat pancreatic acinar cells were incubated with or without CID (25 μM) or CRT (10 μM) for 2 h prior to 100 nM CCK or 200 μM CCh treatment for an additional 30 min. **(A)** IκB-α phosphorylation and degradation were measured in cell cytosolic extract by Western blot. ERK1/2: for loading control. The Western blot results of IκB-α phosphorylation and degradation was quantified and expressed as the ratio of pS IκB-α to IκB-α in the bar figures. **(B)** Western blot analysis of NF-κB P65 in cell cytosolic extract. LDH: for cytosolic extract loading control. The results were representative of two independent experiments.

It has been known that NF-κB is present in the cytoplasm as an inactive state by association with the inhibitory (IκB) proteins. Upon activation, the inhibition of NF-κB by IκBs is removed as soon as IκBs are phosphorylated by IKK kinases and the phosphorylated IκBs rapidly degrade via proteasome-involving pathways. Active NF-κB will redistribute into the nucleus and promote the expression of a number of genes that contain κB-binding sites in their promoters/enhancers (Gukovsky et al., 2003). We here found that PKD activation plays an important role in these events preceding NF-κB activation. Corresponding to PKD inhibition, both IκB-α phosphorylation and degradation caused by CCK- and CCh stimulation were decreased in the cells pre-treated with CID755673 or CRT0066101 (Figure 2A). Further, our Western blot analysis of cell cytosol NF-κB level also showed that either CID755673 or CRT0066101 inhibited nuclear translocation of NF-κB in acinar cells (Figure 2B). The inhibitory effect of the two small compounds on either IκB-α phosphorylation/degradation or nuclear translocation of NF-κB was in accordance with their inhibitory effects on NF-κB-DNA binding activity (Figures 1B,C).

### PKD inhibitors CID755673 and CRT0066101 suppress NF-κB activation in *in vivo* experimental model of pancreatitis induced by cerulein

**Figure 3:**
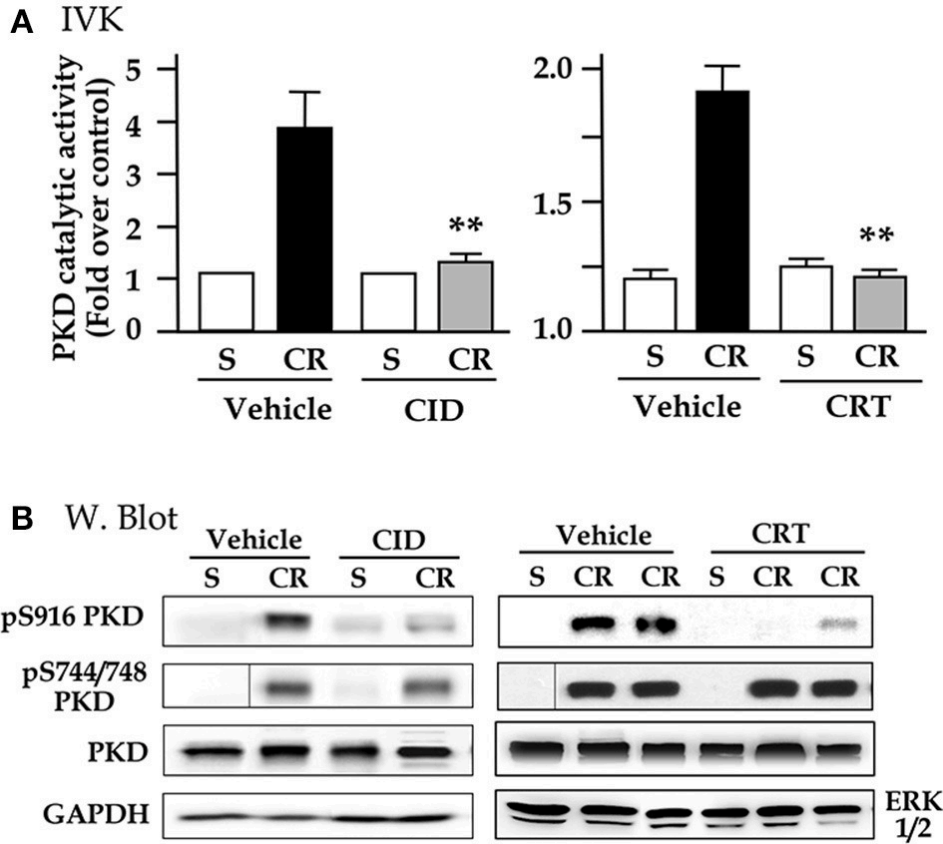
CID755673 or CRT 0066101 pretreatment inhibits PKD activation in cerulein-induced experimental pancreatitis. Rats received intraperitoneal (IP) injection of CID755673 (CID, 15 mg/kg) or CRT 0066101 (CRT, 10 mg/kg). Control rats received same volume of vehicle DMSO. After 60 min waiting time, the inhibitor- or vehicle-pretreated animals were given an IP injection of cerulean (CR) or saline (S). The pancreas was collected in 30 min after the cerulein or saline injection. **(A)** The pancreas tissue lysates were immunoprecipitated with PKD/PKD1 C-20 antibody and PKD1 activity in the immunocomplexes was determined by *in vitro* kinase assay (IVK). PKD/PKD1 catalytic activities were expressed increased fold over control in the animals injected with saline. Results are means ± SE (*n* = 3–5) for each condition. ^**^p < 0.01 vs. cerulein alone without inhibitor pre-treatments. **(B)** Western blot was performed to analyze PKD phosphorylation in the tissue lysates using antibodies against phospho-Ser916 PKD, phospho-Ser744/748 PKD. PKD and GAPDH or ERK1/2: for loading controls. Samples were run in a single gel but were not continuous, as indicated by a line between lanes.

To determine whether the two small molecule PKD inhibitors blocked NF-κB activation in the CCK analog cerulein-induced animal model of pancreatitis, we first tested their *in vivo* selectivity and specificity for PKD when the inhibitors were administered to animals. Rats were IP injected with CID755673 (15 mg/kg) or CRT0066101 (10 mg/kg) and the control rats received the same volume of vehicle (DMSO). Sixty minutes later, cerulein (dissolved in saline) or saline only were administered to the inhibitor- or vehicle-pretreated animals. Thirty minutes after the cerulein injection, we harvested the pancreas and measured pancreatic PKD/PKD1 catalytic activity with *in vitro* kinase assays. As showed in Figure 3A, as high as 60–70% of PKD activation induced by cerulein was inhibited in the animals pretreated with CID755673 or CRT0066101. Similar to the *in vitro* experimental results shown above, the two inhibitors also suppressed PKD Ser916 autophosphorylation (Figure 3B) but had no effect on PKD/PKD1 Ser744/748 phosphorylation which was majorly through PKC-dependent pathways in pancreatitis (Figure 3B), confirming the selectivity and specificity of CID755673 and CRT0066101 targeting PKD *in vivo*.

**Figure 4:**
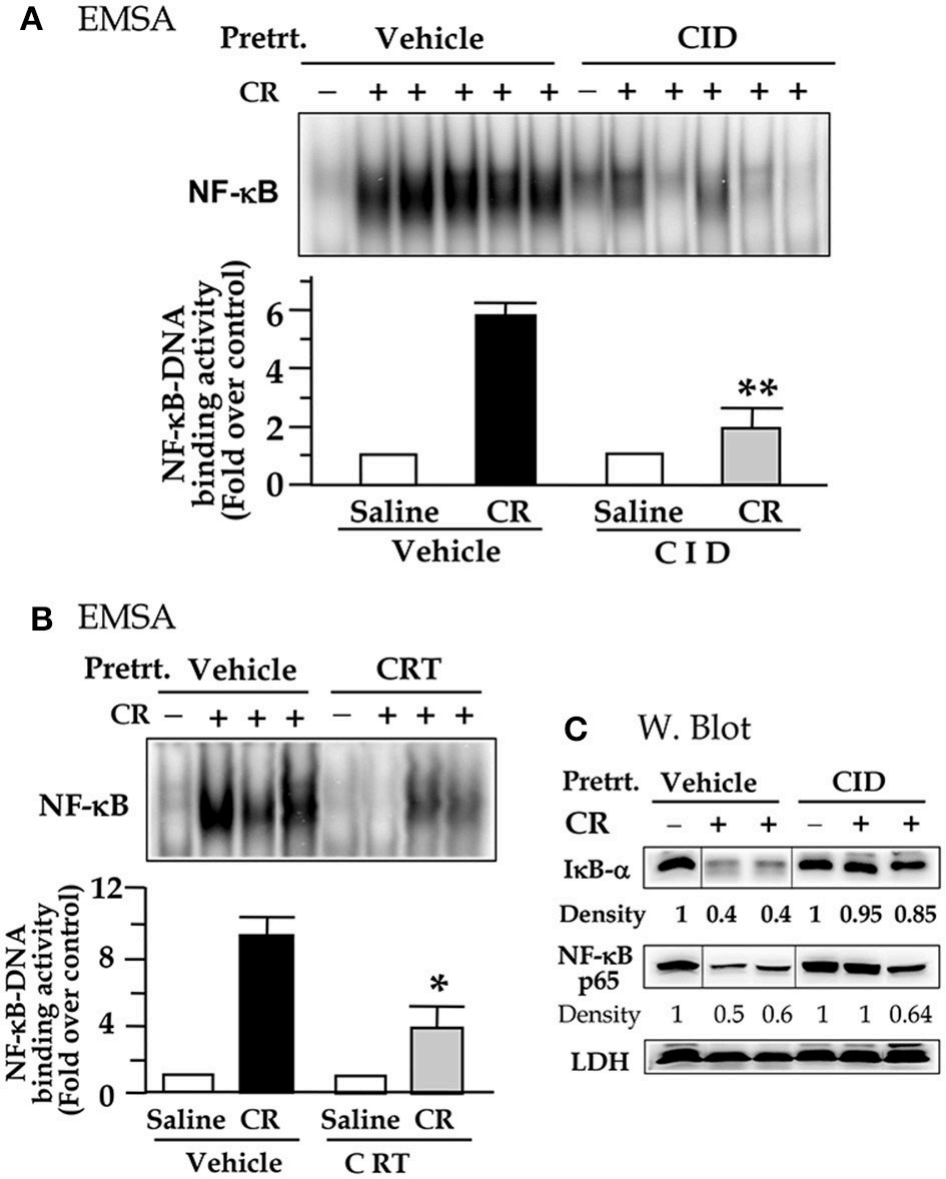
PKD inhibitor CID755673 or CRT 0066101 pretreatment attenuates NF-κB activation in cerulein-induce experimental pancreatitis. Rats received intraperitoneal (IP) injection of CID755673 (CID, 15 mg/kg) or CRT 0066101 (CRT, 10 mg/kg) or same volume of vehicle. After 60 min waiting time, the inhibitor- or vehicle-pretreated animals were given an IP injection of cerulean (CR) or saline (S). The pancreas was collected in 30 min after the cerulein or saline injection. **(A,B)** NF-κB binding activity was measured in pancreatic tissue nuclear extracts by EMSA. Each lane represents one rat. ^**^p < 0.01 or ^*^p < 0.05 vs. cerulein alone without inhibitor pre-treatments. **(C)** IκB-α degradation and nuclear translocation of NF-κB were measured in pancreatic tissue cytosolic extract by Western blot. LDH: for cytosolic extract loading control. Samples were run in a single gel but were not continuous, as indicated by a line between lanes.

We then investigated whether the inhibition of PKD activation by the two small molecule chemicals has effect on NF-κB activation in cerulein pancreatitis animal model. The nuclear extracts prepared from the rat pancreatic tissue were used in EMSA measurement for NF-κB DNA binding activity. Figures 4A,B show that control (saline) rats, treated either without or with either inhibitor, had no or very low NF-κB activity. Cerulein hyperstimulation induced a striking increase in NF-kB activation, which was greatly attenuated (by ~60–70% as quantification in the bar figures of Figure 4) in either CID755673-treated or CRT0066101-treated rats (*P* < 0.01 or *P* < 0.05). The combined results in Figures 3,4 indicate that the inhibition of NF-κB activation by the two inhibitors was closely correlated to their inhibition to PKD activation. Moreover, our Western blot analysis of pancreatic cytosol IκB-α and NF-κB levels showed that CID755673 inhibited degradation of IκB-α and nuclear distribution of NF-κB in cerulein-induced pancreatitis (Figure 4C). The results were consistent with the inhibitory effect of CID755673 on NF-κB activity (Figure 4A).

### NF-κB inhibition by PKD inhibitor CID755673 was associated with decreased pancreatic levels of inflammatory molecules IL-6 and MCP-1

**Figure 5:**
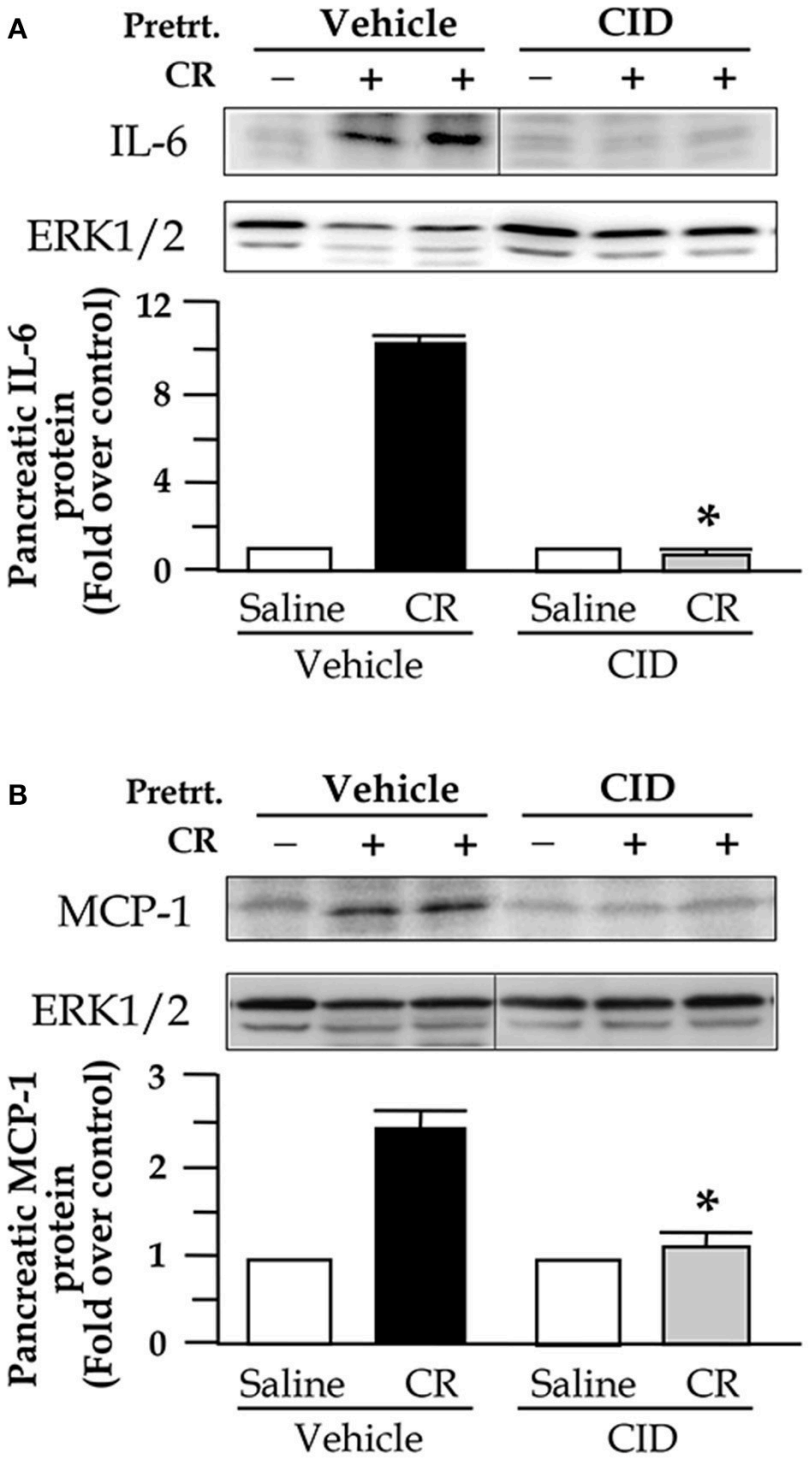
CID755673 (CID) pretreatment decreases pancreatic levels of IL-6 and MCP-1 in cerulein (CR)-induce pancreatitis. Western blot was performed to analyze pancreatic levels of IL-6 and MCP-1 in the tissue lysates using antibody against rat IL-6 **(A)** or MCP-1 **(B)**. ERK1/2: for loading controls. The Western blot band intensities were quantified and normalized by the loading controls and expressed as the ratio over the untreated control in the bar figures. Bar values are means ± SE, ^*^p < 0.05 vs. rats without CID treatments in CR-induced pancreatitis. **(A,B)** Samples were run in a single gel but were not continuous, as indicated by a line between lanes.

To test whether the inhibition of NF-κB by the PKD inhibitor altered expression of inflammatory molecules in pancreatitis, we examined pancreatic level of cytokine IL-6 and chemokine MCP-1. As shown in Figure 5, both IL-6 and MCP-1 levels were very low within the normal pancreas, whereas cerulein induced significantly increased IL-6 and MCP-1 in pancreatitis. CID755673 treatment markedly inhibited pancreatic expression of IL-6 and MCP-1 in cerulein-induced pancreatitis (Figure 5).

### NF-κB inhibition by PKD inhibitor CID755673 was associated with significantly alleviated severity of pancreatitis

Subsequently, we evaluated the effect of NF-κB inhibition by the PKD inhibitor CID755673 on the pathologic changes in pancreatitis. Following 1 h injection of CID755673 (15 mg/kg), we gave the rats up to 4 hourly IP injections of cerulein (20 μg/kg) and the control rats received saline IP injections. The rats were then sacrificed at 30 min and 4 h after the first cerulein injection.

**Figure 6:**
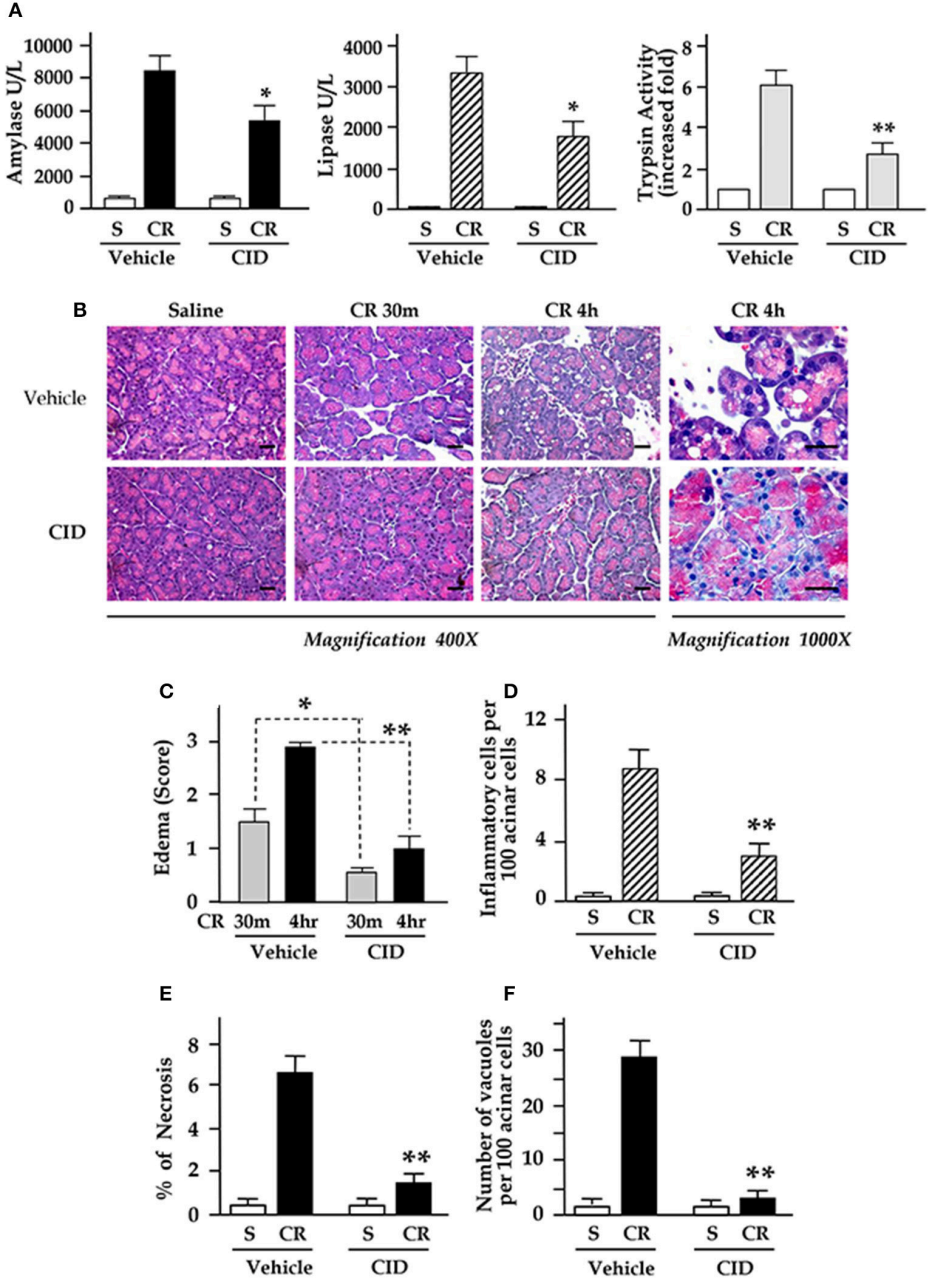
CID755673 (CID) pretreatment attenuates inflammation, necrosis, and severity in cerulein-induced pancreatitis. Rats received IP injection of CID followed by up to 4 hourly IP injections of cerulein (CR) or saline (S), as described in Methods. **(A)** Blood amylase and lipase activation and intrapancreatic trypsin activation (in 30 min after 1st IP injection of CR) in pancreatitis were decreased in CID755673-treated rats. **(B)** H&E stained sections of pancreatic tissue. Edema in early stage (30 min) and late stage (4 h) **(C)** and the percentage of inflammatory cell infiltration **(D)**, necrosis **(E)**, and number of vacuoles **(F)** were measured on H&E stained sections. Values are means ± SE (*n* > 4). ^*^p < 0.05 or ^**^p < 0.01 vs. CR alone without inhibitor pre-treatments.

The results in Figure 6A showed that strikingly increased blood amylase and lipase and intrapancreatic trypsin activation in pancreatitis were significantly decreased in rats with CID755673 pretreatment. CID755673 markedly alleviated the histological injuries in cerulein pancreatitis (Figure 6B). Cerulein-induced interstitial edema was prevented by CID755673 treatment as early as 30 min after 1st injection of cerulein (Figures 6B,C). Furthermore, the major pancreas histological changes in animals pretreated with CID755673 are the strikingly attenuation of inflammatory cell infiltration and acinar cell necrosis in pancreatitis (Figures 6D,E). Accumulation of cytoplasmic vacuoles was also significantly reduced in CID755673-treated rats (Figure 6F). These results achieved from the *in vivo* animal model of pancreatitis indicated that NF-κB inhibition by the PKD inhibitor CID755673 was associated with significantly decreased severity of acute pancreatitis.

### Therapeutic benefits of novel small molecule PKD inhibitor in rodent experimental pancreatitis

**Figure 7:**
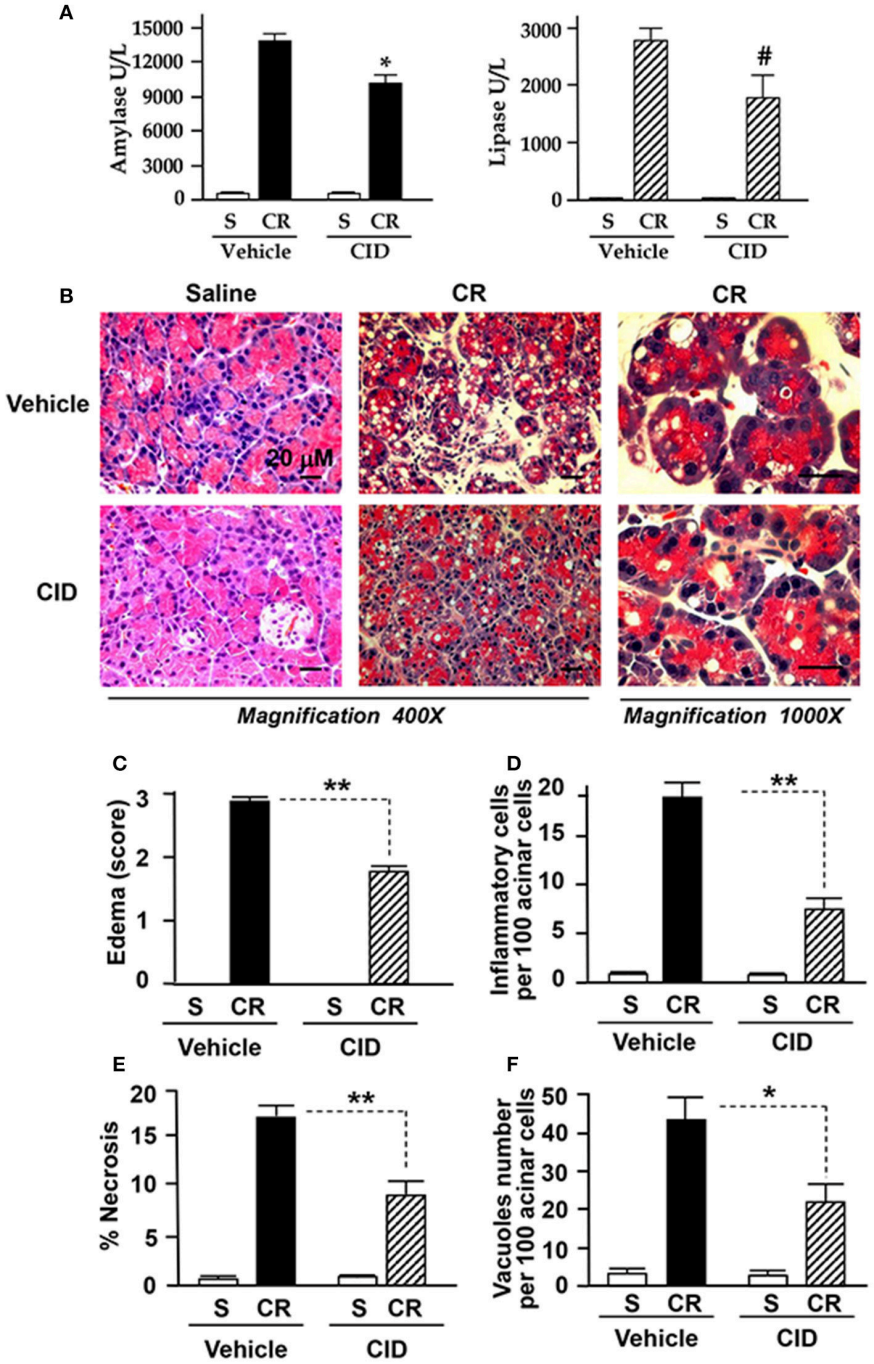
CID755673 post-treatment ameliorated the severity of pancreatitis. **(A)** Blood amylase and lipase activation was decreased in cerulein-induced rat pancreatitis treated with PKD inhibitor CID755673 (CID, 15 mg/kg). **(B)** Representative H&E-stained pancreatic sections from rat pancreatitis treated without or with CID 755673 compound. Compared to rats without CID755673 treatment, cerulein (CR) induced pancreatitis was greatly ameliorated in rats treated with CID755673 (CID). Edema **(C)** and the percentage of inflammatory cell infiltration **(D)** necrosis **(E)** and number of vacuoles **(F)** were measured on H&E stained sections. Values are means ± SE (*n* > 3). ^*^p < 0.05, ^**^p < 0.01, or #*p* = 0.054 vs. CR alone without inhibitor pre-treatments.

To determine potential of PKD inhibitors as therapeutics after the disease has commenced, we administered CID755673 (15 mg/kg) or vehicle by intraperitoneal (IP) injection to rats in early and late stages after the initiation of pancreatitis (i.e., 30 min after 1st and 4th of IP injection of cerulein). The animals were sacrificed in 1 h after the 6th hourly cerulein injections and the pancreas were collected. We found that blood amylase and lipase dramatically elevated after 6 hourly IP injections of cerulein (Figure 7A), but, both enzymes were markedly decreased in CID755673-treated rats although the change of lipase did not have statistical significance (*P* = 0.054) between CID755673-treated and the control groups (Figure 7A). Histology staining of the pancreatic tissue indicated that treatment of CID755673 obviously improved pancreatic histopathology (Figure 7B). Compared to vehicle-treated rats, there was 50–60% reduction of pancreatic edema (Figure 7C), inflammatory cell infiltration (Figure 7D), necrosis (Figure 7E), and vacuole accumulation (Figure 7F) in CID755673-treated rodents after 6 hourly cerulein injections.

## Discussion

Acute pancreatitis is a serious medical disorder and at present, there are no treatments targeting its molecular pathogenesis. The transcription factor NF-κB has been known as an important regulator of the production of the inflammatory molecules and plays a critical role in the inflammatory response and parenchymal injury in pancreatitis (Gukovsky et al., 1998, 2003; Frossard et al., 1999; Satoh et al., 1999; Tak and Firestein, 2001; Vaquero et al., 2001; Chen et al., 2002). NF-κB activation is an early event in experimental pancreatitis, thus blocking the upstream signaling pathways mediating NF-κB activation in pancreatitis will be important for early treatment of this serious disease.

Our previous studies with molecular approach in AR42J cell line showed that PKD was required in NF-κB activation in *in vitro* model of pancreatitis induced by CCK and the cholinergic agonist CCh (Yuan et al., 2008), it is therefore of significance to further investigate the function of PKD in NF-κB activation with *in vivo* experimental models of pancreatitis. Recent development of novel small molecule chemical PKD inhibitors brings us a useful pharmacological approach to achieve this goal. We report here that PKD is a critical regulator of NF-κB activation in experimental models of pancreatitis. Either *in vitro or in vivo* application of the two PKD inhibitors, CID755673 and CRT0066101, significantly prevented NF-κB activation in early stage of pancreatitis.

We first showed that either CID755673 or CRT0066101 was a specific PKD inhibitor that potently blocked agonist-induced PKD autophosphorylation at Ser916 without affecting PKC-dependent Ser744/748 phosphorylation (that was inhibited by PKC inhibitor Go6983, Figure 1). More important finding was that PKD inhibition resulted in markedly suppressed NF-κB-DNA binding activity in nuclear extracts from the acini pretreated with CID755673 or CRT0066101 (Figure 1). The results confirm the requirement of PKD in NF-κB activation previously reported by our studies on AR42J cells with a molecular approach (Yuan et al., 2008).

It has been known that NF-κB activation takes place when the inhibitory IκB protein is degraded and dissociated with the NF-κB complex. Activated NF-κB then redistributes to nucleus and promotes the expression of a number of genes that contain κB-binding sites in their promoters (Gukovsky et al., 2003; Yuan et al., 2008). We examined the effect of the two PKD inhibitors on IκB-α protein and NF-κB nuclear translocation. CCK or CCh hyperstimulation caused IκB-α phosphorylation/degradation and nuclear translocation of NF-κB in the pancreatic acini (Figure 2). These events were potently inhibited by treatment of either CID755673 or CRT0066101, whereas the inhibitor did not change IκB-α and cytosol NF-κB amount in control cells (Figure 2). These results indicate that the PKD inhibitors attenuate pancreatic NF-κB activation and subsequent nuclear translocation by preventing the phosphorylation and degradation of IκB protein. PKD has been reported to mediate NF-κB activation at the level of the IKK complex in oxidative stress. PKD could directly interact with IKK, leading to its activation (Storz et al., 2004b). Our evidence presented here also suggests that PKD mediates NF-κB activation through acting at targets upstream of IκB degradation, probably through upstream of IKK kinases. The detail mechanisms remain to be further illustrated.

Next, we employed CID755673 and CRT0066101 in animals to study whether PKD inhibition affected NF-κB activation in *in vivo* experimental model of pancreatitis. Consistent with our *in vitro* experimental results, selective and specific inhibition of PKD catalytic activity and Ser 916 autophosphoryltion by either inhibitor (Figure 3) resulted in strikingly decreased NF-κB activation in early stage of acute pancreatitis in rats pre-treated with CID755673 or CRT0066101 compound (Figure 4).

We then explored whether suppressing NF-κB activation by the PKD inhibitors was associated with attenuation of inflammatory responses and severity of the disease in experimental model of pancreatitis. We found that NF-κB inhibition by CID755673 was associated with significantly decreased inflammatory responses indicated by reduced pancreatic IL-6 and MCP-1 proteins (Figure 5), less inflammatory cell infiltration in pancreatic tissue sections, and reduced pancreatic trypsin activation, necrosis, edema and vacuolization (Figure 6).

Cytokines have multiple functions in regulating the immunoinflammatory responses. In particular, IL-6 plays a key role in the convert from neutrophils to macrophages during the transformation from innate to acquired immunity (Maianski et al., 2004; Jones, 2005; Gukovsky et al., 2008). Of significance for pancreatitis is that a good correlation between the level of IL-6 with the disease severity has been observed in human pancreatitis (Bhatia et al., 2000). MCP-1 is a strong chemoattractant for the recruitment of monocytes/macrophages (Maianski et al., 2004; Jones, 2005; Gukovsky et al., 2008). During pancreatitis, not only the inflammatory cells but also injured acinar cells can produce MCP-1 (Blinman et al., 2000; Bhatia et al., 2005; Gukovsky et al., 2008). Further evidence showed that inhibition of MCP-1 resulted in amelioration of both acute cerulein pancreatitis (Bhatia et al., 2005) and the chronic pancreatitis induced by dibutyltin dichloride (Zhao et al., 2005).

The transcription factor NF-κB has been known as a major regulator of the inflammatory molecules expression in pancreatitis. Both IL-6 and MCP-1 expression is under the control of NF-κB because promoters of these genes have DNA binding sites of NF-κB (Ben-Baruch et al., 1995; Marks-Konczalik et al., 1998). In many inflammatory diseases including pancreatitis, activated NF-κB translocates to neucleus and binds to the gene promoters for proinflammatory cytokine IL-6 and monocyte chemoattractant MCP-1, resulting in increased production of IL-6 and MCP-1 (Martin et al., 1999; Pandol et al., 1999; Roebuck et al., 1999; Tak and Firestein, 2001; Vaquero et al., 2001; Gukovsky et al., 2008). Therefore, we here examined IL-6 or MCP-1 protein level in pancreatic tissue lysates and found that NF-κB inhibition by PKD inhibitor CID755673 was associated with significantly reduced pancreatic production of IL-6 and MCP-1 proteins. The results suggest that PKD inhibitor could suppress expression of the inflammatory molecule IL-6 and MCP-1 through inhibiting NF-κB activation in pancreatitis.

Previously, we investigated whether the PKD inhibitor CRT0066101 would prevent zymogen activation in pancreatitis in an *in vitro* pancreatitis model in rat pancreatic acinar cells (Thrower et al., 2011). We found that secretagogue-induced zymogen activation was markedly reduced by treatment of the acini with the PKD inhibitor without affecting basal zymogen activation or secretion. Particularly, trypsin activities were all dramatically reduced in CCK-, CCh-, and bombesin-stimulated cells which had been pretreated with the PKD inhibitor CRT0066101. Further studies showed that CCK-induced cathepsin B activity was inhibited by CRT0066101 (Thrower et al., 2011). These results indicated that the potential mechanism by which PKD mediates zymogen activation was through regulating cathepsin B, a lysosomal hydrolase that has been known to play a role in intrapancreatic trypsinogen activation and the onset of acute pancreatitis. Of significance, we here demonstrated that PKD inhibitor also strongly inhibited intrapancreatic trypsin activation and pancreatic vacuolization in *in vivo* rat cerulein-induced pancreatitis, which supported our previous *in vitro* experimental results, confirming again PKD regulates zymogen activation in pancreatitis.

There was very little information regarding application of the PKD inhibitors in therapy of pancreatitis in experimental animal models. It is exciting that our final results achieved from experiments in which the rats were treated by CID755673 after the pancreatitis has commenced showed that the post-treatment of the PKD inhibitor significantly attenuated pancreatic injury in cerulein-induced pancreatitis (Figure 7). The studies further confirmed that PKD is an important molecular target for therapy of acute pancreatitis and suggest a high therapeutic potential of the small molecule PKD inhibitors in this disorder.

**Figure 8:**
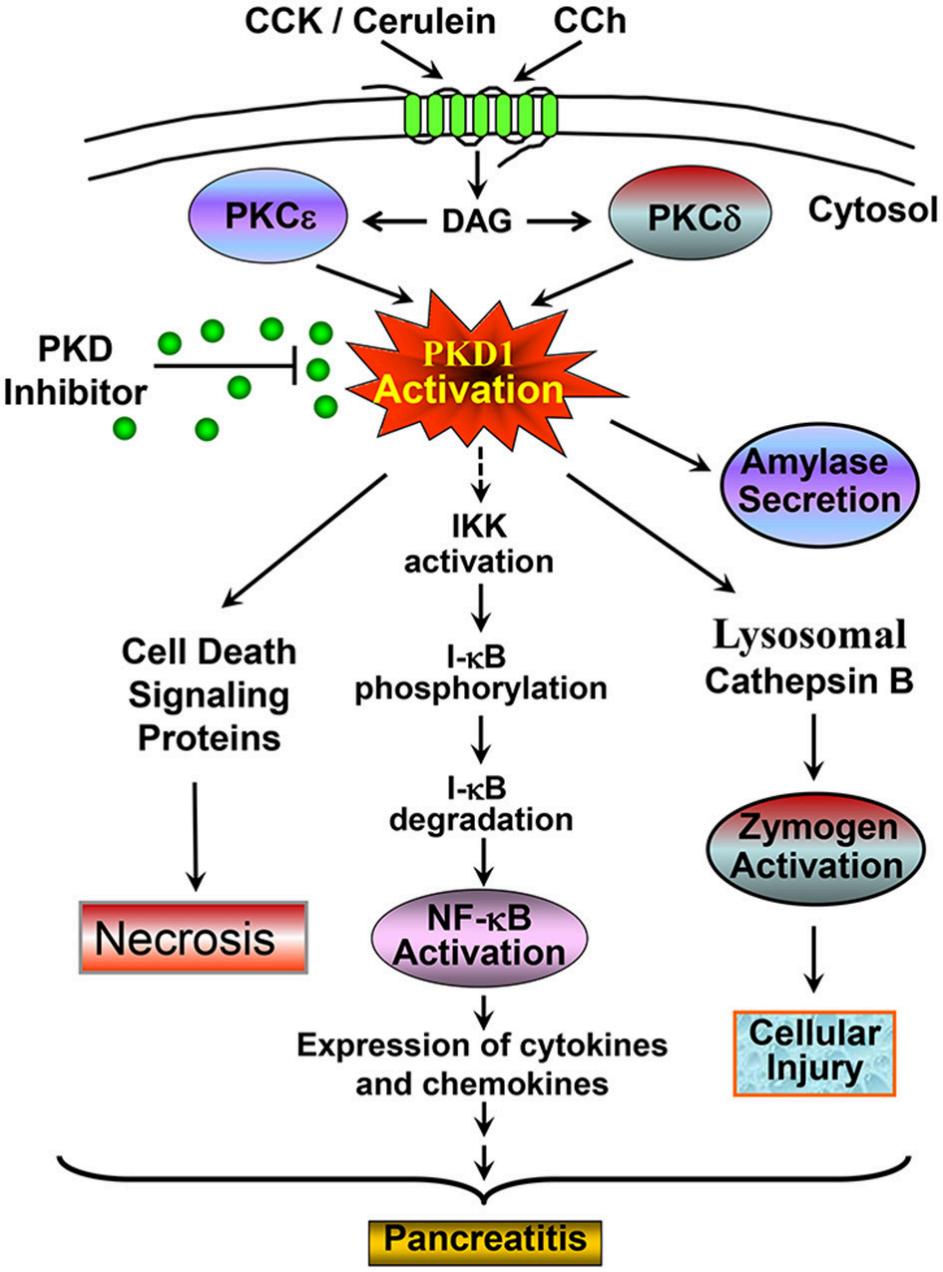
A scheme summarizing the signaling pathway through which PKD mediates NF-κB activation and the role of PKD in multiple pathological processes associated with pancreatitis. Our previous studies identified PKD as a convergent point for PKCδ and ε activation in the signal pathways initiated through CCK or CCh G-protein receptors and mediated NF-κB activation in pancreatic acinar cells (Yuan et al., 2008). Here with both *in vitro* and *in vivo* rat pancreatitis models, we found that activated PKD regulates NF-κB activation in CCK/cerulein-induced pancreatitis through promoting IκB protein phosphorylation and degradation. Activation of NF-κB results in inflammation and pancreatitis. Small molecule PKD inhibitors can block this pathobiologic process. This scheme also summarized the role of PKD in multiple pathological processes associated with pancreatitis. CCK/cerulein-induced PKD activation promotes necrosis in pancreatitis by regulating multiple cell death signaling proteins (Yuan et al., 2012); Active PKD also mediates amylase secretion and zymogen activation through regulating cathepsin B (Thrower et al., 2011). These pathologic responses can be blocked when PKD is inhibited by small molecule PKD inhibitors, resulting in amelioration of the severity of pancreatitis. All these effects indicate that PKD may represent a potential therapeutic target in pancreatitis. See the text and references (Yuan et al., 2008, 2012; Thrower et al., 2011) for details.

In summary, our studies demonstrate that PKD is a key mediator of NF-κB activation in *in vivo* model of pancreatitis. Small chemical inhibitors of PKD have significant potential as therapeutic intervention for pancreatitis through blocking NF-κB activation, a critical early pathological event in this disease. Combining with our previous reports (Yuan et al., 2008, 2012; Thrower et al., 2011; Yuan and Pandol, 2016) that identified PKD as an early convergent target of PKCδ and ε signaling in NF-κB activation in exocrine pancreas, we summarized our findings in Figure 8 to show the signaling pathways of PKD mediating NF-κB activation in pancreatitis. In addition, we here also presented/summarized the overall role of PKD, which we found so far, in multiple pathological processes associated with pancreatitis (Figure 8).

## Author contributions

JY drafted the manuscript and designed the study. JY, TT, MG, GT, and CC acquired and analyzed the data. JY and SP interpreted the data, critically revised the manuscript, and approved the version to be published.

### Conflict of interest statement

The authors declare that the research was conducted in the absence of any commercial or financial relationships that could be construed as a potential conflict of interest.
